# SELF-Former: multi-scale gene filtration transformer for single-cell spatial reconstruction

**DOI:** 10.1093/bib/bbae523

**Published:** 2024-10-16

**Authors:** Tianyi Chen, Xindian Wei, Lianxin Xie, Yunfei Zhang, Cheng Liu, Wenjun Shen, Si Wu, Hau-San Wong

**Affiliations:** Department of Computer Science, City University of Hong Kong, Kowloon 999077, Hong Kong; Department of Computer Science, City University of Hong Kong, Kowloon 999077, Hong Kong; School of Computer Science and Engineering, South China University of Technology, Guangdong 510006, China; School of Future Technology, South China University of Technology, Guangdong 511442, China; Department of Computer Science, Shantou University, Shantou 515063, China; Department of Bioinformatics, Shantou University Medical College, Shantou 515041, China; School of Computer Science and Engineering, South China University of Technology, Guangdong 510006, China; Department of Computer Science, City University of Hong Kong, Kowloon 999077, Hong Kong

**Keywords:** single-cell RNA sequence, spatial transcriptomics, transformer, multi-scale, gene filtration

## Abstract

The spatial reconstruction of single-cell RNA sequencing (scRNA-seq) data into spatial transcriptomics (ST) is a rapidly evolving field that addresses the significant challenge of aligning gene expression profiles to their spatial origins within tissues. This task is complicated by the inherent batch effects and the need for precise gene expression characterization to accurately reflect spatial information. To address these challenges, we developed SELF-Former, a transformer-based framework that utilizes multi-scale structures to learn gene representations, while designing spatial correlation constraints for the reconstruction of corresponding ST data. SELF-Former excels in recovering the spatial information of ST data and effectively mitigates batch effects between scRNA-seq and ST data. A novel aspect of SELF-Former is the introduction of a gene filtration module, which significantly enhances the spatial reconstruction task by selecting genes that are crucial for accurate spatial positioning and reconstruction. The superior performance and effectiveness of SELF-Former’s modules have been validated across four benchmark datasets, establishing it as a robust and effective method for spatial reconstruction tasks. SELF-Former demonstrates its capability to extract meaningful gene expression information from scRNA-seq data and accurately map it to the spatial context of real ST data. Our method represents a significant advancement in the field, offering a reliable approach for spatial reconstruction.

## Introduction

Single-cell RNA sequencing (scRNA-seq) technology enables the measurement of gene expression in individual cells across millions of samples, providing detailed insights into complex cellular landscapes and heterogeneity [1]. The task of recovering spatial positions can provide a new spatial perspective for scRNA-seq [2, 3]. This is because scRNA-seq methods involve dissociating tissues into suspensions at single-cell resolution, leading to the loss of crucial information regarding spatial localization. Such spatial information is vital for understanding biological processes, including cell spatial annotation [4, 5], cell states [6, 7], and cell trajectories [8, 9]. Accurate reconstruction is essential for gaining comprehensive insights into the spatial organization of cellular components within tissues, facilitating a deeper understanding of intricate biological phenomena.

In recent years, spatial transcriptomics (ST) has gained increasing popularity due to its capability of capturing the spatial distribution of gene expression in tissues at high-resolution [10, 11]. However, it comes with drawbacks, including high costs and reduced gene detection performance at single-cell resolution compared to scRNA-seq methods, particularly for weakly-expressed genes [12, 13]. Nevertheless, the emergence of ST has paved the way for the potential alignment of scRNA-seq data [14]. This facilitates knowledge transfer between the two data modalities, enabling the recovery of inherent spatial properties in scRNA-seq data. The adoption of effective methods for the alignment of ST features associated with scRNA-seq data has significantly improved the accuracy of spatial position recovery, while reducing manual effort, resources, and computational costs at the same time [15, 16].

The key to understanding scRNA-seq data as a reconstruction task lies in recognizing that, while scRNA-seq provides high-resolution information on individual cell gene expression, it lacks the actual spatial location information of cells within tissues. An important objective of our work is to recover the spatial distribution of cells in tissues or organs based on scRNA-seq data, enabling a more comprehensive understanding of tissue structure and cell interactions. This is crucial for unveiling biological processes, studying tissue development and comprehending disease mechanisms. As a result, a correct understanding and effective processing of scRNA-seq data for reconstruction will facilitate in-depth exploration of cell spatial distribution and interactions within tissues.

Early research typically used the construction of mapping matrices to establish associations between scRNA-seq and ST data, in order to obtain the positional attributes corresponding to scRNA-seq data. For instance, SpaOTsc [16] has devised an optimal transport algorithm [17] to measure the distance between two cellular data modalities for reconstruction. Previous methods [18, 19] focused on using mapping matrices to establish the correspondence between cells. These methods face notable limitations. First, the correspondence between scRNA-seq and ST data is inherently complex, and existing mapping matrices often fail to capture this complexity effectively. These matrices typically assume that the number of cells or spots is less than the number of genes, which in real datasets, leads to the mapping matrix establishing connections between cells and spots that do not accurately reflect biological reality. This misalignment causes the method to overly focus on the association between cells and spots, while overlooking critical interactions between genes. Furthermore, the contribution of each gene in the reconstruction task varies, current approaches do not adequately account for the distinct roles of different genes in the reconstruction task. Therefore, it is necessary to develop more advanced methods to manage the complex relationship between scRNA-seq data and ST data. This will not only improve the accuracy of data integration but also provide a more comprehensive understanding of the functions and mechanisms within biological systems.

To address these challenges, we employed the transformer structure for modeling reconstruction task, and propose a single-cell multi-scale gene filtration transformer (SELF-Former) to accurately reconstruct the spatially resolved scRNA-seq data. Specifically, we introduce a self-attention mechanism in scRNA-seq data, which emphasizes the interaction between cells to capture the feature transformation from non-spatially resolved scRNA-seq data to spatially resolved ST data. For each transformer block, we introduced a gene-based filtration learning module by modifying the attention mechanism. It automatically filters out genes with high contribution and filters out genes with low contribution, thereby improving the model’s reconstruction performance for ST data. In addition, to address the problem of lack of spatial location in scRNA-seq data, we combined spatial correlation regularization to further impute genes for scRNA-seq data. The proposed SELF-Former eliminates the need for additional tuning techniques, enabling seamless training and testing across benchmarks. Subsequently, we further demonstrate the biological analysis of SELF-Former, such as the accuracy and efficiency of gene filtration, removal of batch effects and performance evaluation.

## Materials and methods

### Methods

**Figure 1:**
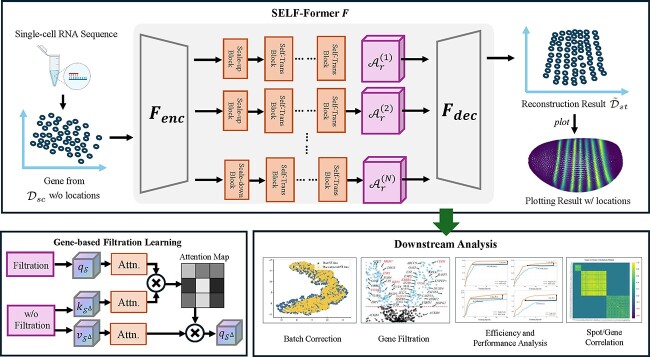
The flowchart of proposed SELF-Former $F$. We take a random gene from the drosophila as an example: firstly, high-throughput expression data for the gene is extracted from single-cell resolution, as shown in the left image which lacks spatial information. Secondly, the scRNA-seq data is encoded from $F_{enc}$, primarily implemented as a self-attention transformer block. The bottom left corner illustrates an overview of gene-wise filtration learning. Next, features are aggregated along multi-scale self-attention blocks to obtain spatially resolved predictions of ST data. The aggregated representations are then processed through the decoder $F_{dec}$. The decoder reconstructs the ST data from these multi-scale features. The final output is the reconstructed ST data, which includes spatial locations for each gene expression, allowing for spatial plotting and further downstream analysis.

In this section, we present the details of the proposed method, which aims to spatially recovering scRNA-seq data by assigning spatial location for each cell. Figure 1 provides an overview of SELF-Former for spatially alignment scRNA-seq data.

#### Problem Formulation

Here, we first give some notations. We are provided with scRNA-seq data $\mathcal{D}_{sc}=\{(x_{i})_{i=1}^{n_{sc}} \in \mathbb{R}^{\mathcal{C}_{p}} \}$ and ST data $\mathcal{D}_{st}=\{(x_{i})_{i=1}^{n_{st}} \in \mathbb{R}^{\mathcal{C}_{q}} | (y_{i})_{i=1}^{n_{st}} \in \mathbb{R}^{2}\}$. Here, $n_{sc}$ and $n_{st}$ represent the number of cells in scRNA-seq data and spots in ST data, respectively. And notations $\mathcal{C}_{p}$ and $\mathcal{C}_{q}$ denote the total number of genes measured in raw count scRNA-seq and ST data. Typically, $\mathcal{C}_{q} \subset \mathcal{C}_{p}$ and $\mathcal{C}_{p} \gg \mathcal{C}_{q}$, as scRNA-seq assays many genes without defined spatial patterning in the tissue. We need to establish a framework for reconstruction, aiming to extract commonalities in the data from two cellular modalities. Overlapping genes, denoted as $\mathcal{C}_{o} = \mathcal{C}_{q} \cap \mathcal{C}_{p}$, represent the subset of genes that are present in both scRNA-seq and ST data. These genes are biologically significant because they provide a common ground for comparing and aligning the two datasets. By focusing on these shared genes, we ensure that the reconstructed ST data leverages the most relevant and consistent gene expression information from both modalities. This reduces noise and potential biases introduced by genes that are only present in one dataset but not the other. Therefore, by normalizing and subsetting to these $\mathcal{C}_{o}$ overlapping genes, the raw count data can be rewritten as scRNA-seq data $\mathcal{D}_{sc}=\{(x_{i})_{i=1}^{n_{sc}} \in \mathbb{R}^{\mathcal{C}_{o}} \}$ and predicted ST data $\tilde{\mathcal{D}}_{st}=\{(\tilde{x_{i}})_{i=1}^{n_{st}} \in \mathbb{R}^{\mathcal{C}_{o}} | (y_{i})_{i=1}^{n_{st}} \in \mathbb{R}^{2} \}$, $(y_{i})_{i=1}^{n_{st}} $ denotes the spatial location matrix of each cell in the ST, while $(y_{i})_{i=1}^{n_{sc}} $ for the scRNA-seq data is unknown. The key to this problem is how to accurately impute scRNA-seq data to corresponding ST data while preserving spatial positional relationships.

#### Design of backbone

The abundant gene expression data obtained through scRNA-seq presents a challenge in establishing the connection between scRNA-seq and ST data for reconstruction tasks. To address this, we have considered the attention mechanism within the transformer structure. By employing the key-query-value composition of the self-attention mechanism commonly used in computational tasks, we aim to identify rich gene expression patterns among scRNA-seq data, thus bridging the gap for reconstruction of ST data. Before delving further into this approach, let’s briefly review the transformer structure: for query, key and value $\{ Q, K, V\}$, we employ fully connected layers to learn non-linear feature expressions, formulated as follows: 


(1) \begin{align*}& \{ Q, K, V\} = \oslash (W_{\{q, k, v\}} \otimes \mathcal{D}_{sc} + b_{\{ q, k, v\}}),\end{align*}

where the $W_{\{q, k, v\}}$ and $b_{\{ q, k, v\}}$ are learnable model parameters, and $\oslash $ and $\otimes $ represent the LeakyReLU activation function and matrix multiplication. In above equation, we employ a self-attention mechanism to learn the query, key and value for scRNA-seq data. Allowing the network to utilize the rich gene expression from scRNA-seq data to learn intrinsic expression patterns. The output at each position represents the sum of values weighted by the scaled dot-product similarity between the keys and queries. The equation is formulated as follows: 


(2) \begin{align*}& \mathcal{A} = \mathit{\text{Softmax}} (Q \otimes K / \sqrt{\Lambda}) \otimes V,\end{align*}

where $\Lambda $ denotes the number of feature channels. To understand this in a biological context, we interpret the components of this equation as follows. Query ($Q$) and Key ($K$) Matrices are derived from the scRNA-seq data, representing gene counts. The multiplication $Q \otimes K$ captures the interactions between different genes. In a biological sense, this can be seen as quantifying the potential relationships and dependencies among genes based on their expression levels. Softmax Normalization: the softmax function applied to $Q \otimes K / \sqrt{\Lambda }$ normalizes the interaction scores to a probability distribution, which we refer to as the self-attention matrix. This matrix highlights the associations between genes, assigning higher weights to more significant interactions. Biologically, this means identifying which genes are more likely to influence each other. Value Matrix ($V$) is also derived from the scRNA-seq data and contains the actual expression values. The multiplication of the attention matrix with $V$ results in $\mathcal{A}$, where the gene expression values are weighted by their learned importance, thus emphasizing the most biologically relevant gene expressions for the target ST data. The self-attention mechanism allows our model to dynamically focus on different genes and their interactions at each layer. By stacking multiple self-attention blocks, the model progressively refines its understanding of the gene expression landscape, incorporating both local and global gene interactions. To maintain the integrity of the input features across layers, we introduce residual connections in each self-attention block. This ensures that the original gene expression information is preserved and that the associations between the predicted ST data and scRNA-seq data remain intact throughout the network. Meanwhile, we believe that the input features to the self-attention block should be controllable. We apply an adaptive factor denoted as $\lambda $ to adjust the influence of input data on the output features of the attention block and the Equation 2 can be rewritten as follows: 


(3) \begin{align*}& \mathcal{A}_{r} = \text{softmax} (Q \otimes K / \sqrt{\Lambda}) \otimes V + \lambda \mathcal{D}_{sc}.\end{align*}

Different scales of gene expression information are equally important. To address this, we have designed attention modules at different scales, with corresponding intermediate feature dimensions set to 1024, 2048, and 4096. With the aggregation of multiple transformer blocks, the proposed SELF-Former $F$ explores the interdependence of gene expression across various receptive fields. The output of multiple residual blocks is then concatenated together to build the final output $\mathcal{A}_{agg}$ as follows: 


(4) \begin{align*}& \mathcal{A}_{agg} = \text{concat} (\mathcal{A}_{r}^{(1)},..., \mathcal{A}_{r}^{(N)}),\end{align*}

where $N$ represents the number of scales, typically set to 3. The SELF-Former employs two distinct modeling approaches for spatial reconstruction. One is the global relationship modeling. SELF-Former approach captures broad correlations between gene expressions, ensuring that the model understands the overall gene expression landscape. The other one is local relationship modeling. Within each module stack, local modeling reshapes the scRNA-seq data itself. By learning from the attention matrix, the model captures finer, intrinsic correlations between genes, enhancing its ability to reconstruct spatial data accurately. Overall, the self-attention mechanism and multi-scale modules enable our model to effectively capture and interpret complex gene expression patterns, leading to accurate spatial reconstruction. This biologically informed approach ensures that the intrinsic relationships between genes are preserved and leveraged throughout the modeling process.

The input data $\mathcal{D}_{sc}$ undergoes the model to obtain predictions, and we impose a mean squared error constraint formulated as follows: $\mathcal{L}_{recon} = \mathbb{E}_{F}[|| \mathcal{D}_{st} - F(\mathcal{D}_{sc}) ||_{2}^{2}]$. Further, considering the prevalence of zero values in different datasets. It indicates a considerable sparsity in both scRNA-seq and ST data. In light of this observation, we devise an binary mask $\mathcal{M} \in \mathbb{R}^{0, 1}$ to record the positions of gene expression in the reference ST data $\mathcal{D}_{st}$ where values are zero. The formulation is as follows: 


(5) \begin{align*}& \mathcal{L}_{mask} = \mathbb{E}_{F}[|| \mathcal{M} \odot (\mathcal{D}_{st} - F(\mathcal{D}_{sc})) ||_{2}^{2}],\end{align*}

where notation $\odot $ represents the element-wise matrix multiplication. By minimizing the aforementioned mean squared error loss $\mathcal{L}_{recon}$, SELF-Former achieves a global estimation of the predicted output. And simultaneously employing $\mathcal{L}_{mask}$ to facilitate the estimation of non-zero elements, the SELF-Former is trained to impute the spatial locations of scRNA-seq data. This enhances the model encoding ability for scRNA-seq data in both intrinsic and extrinsic gene expression patterns, thereby improving the integration predictions of spatially resolved reference data.

However, due to the lack of constraints on the gene expression level, such predictions do not align well with the target spatial data distribution. Inspired by the Pearson correlation coefficient, we designed a gene-wise correlation constraints to regularize the correlation of gene expression for each spatial data column, thereby imposing constraints on the overall gene expression. Here, we first present the formula for the Pearson correlation coefficient: 


(6) \begin{align*}& \rho(\tilde{\mathcal{D}}_{st}, {\mathcal{D}}_{st}) = \frac{\text{Cov}(\tilde{\mathcal{D}}_{st}, {\mathcal{D}}_{st})}{\sigma_{\tilde{\mathcal{D}}_{st}} \sigma_{{\mathcal{D}}_{st}}},\end{align*}

where $\text{Cov}$ denotes the covariance and $\sigma $ represents the standard deviation. Considering that the gene expression in each column $j$ needs to be constrained with reference to the spatial data, we further designed the gene-wise correlation loss: 


(7) \begin{align*}& \mathcal{L}_{corr} = \mathbb{E}_{F} [1 - \frac{1}{n} \sum_{j=1}^{n} \rho(\tilde{\mathcal{D}}_{st}^{:,j}, {\mathcal{D}}_{st}^{:,j})].\end{align*}

Minimizing the loss function ensures that the latter term in the Equation 7 approaches to $1$, indicating that the gene-wise correlation approximates the expression of the reference ST data. The comprehensive optimization formulation, recovering all the aforementioned regularization methods is represented as follows: 


(8) \begin{align*}& \min_{\theta_{F}} \mathcal{L}_{recon} + \alpha_{1} \mathcal{L}_{mask} + \alpha_{2} \mathcal{L}_{corr},\end{align*}

where hyper parameters $\alpha _{1}$ and $\alpha _{2}$ quantify the impact of each loss function on the final optimization objective.

#### Gene-based filtration learning

The model receives input scRNA-seq data with abundant gene expressions and the dimension of $n_{o}$ is relative high. However, among the vast number of genes, useful genes for reconstruction have yet to be explored. For example, marker genes in the dataset are more likely to aid in the imputation of spatial information. While certain genes, such as other thyroid-related genes may have a negative impact on reconstruction. Based on this observation, we propose a gene-based filtration learning designed to filter individual positive genes from the high-dimensional gene set for subsequent predictions. Single negative genes are omitted to prevent their influence on the final predictions.

We define the matrix resulting from dividing the query $Q$ and key $K$ in the self-attention mechanism and then applying the softmax as the gene correlation matrix $\mathcal{S}$, whose size is $ [C_{o}, C_{o}]$. Each element on the diagonal of matrix $diag(\mathcal{S})$ represents the contribution of an individual gene to the Value $V$. We sort the contributions in descending order and employ an adaptive filtration factor $\phi \in [0, 1]$ for gene selection. When $\phi = 0.5$, it selects 50% of the effective elements. Consequently, the shape of the filtration matrix $\mathcal{S}^{\triangle }$ is $[C_{o} // 2, C_{o} // 2]$. Subsequently, we apply the filtration matrix $\mathcal{S}^{\triangle }$ to the input data $\mathcal{D}_{sc}$ to obtain the gene expression matrix $\mathcal{D}_{{sc}^{\triangle }}$ with $C_{o} // 2$ genes. We use the filtration matrix as the key $K_{\mathcal{S}^{\triangle }}$ and value $V_{\mathcal{S}^{\triangle }}$ in the self-attention mechanism. The formulation is as follows: 


(9) \begin{align*}& \{ K_{\mathcal{S}^{\triangle}}, V_{\mathcal{S}^{\triangle}}\} = \oslash (W_{\{k_{\mathcal{S}^{\triangle}}, v_{\mathcal{S}^{\triangle}}\}} \otimes \mathcal{D}_{{sc}^{\triangle}} + b_{\{ k_{\mathcal{S}^{\triangle}}, v_{\mathcal{S}^{\triangle}}\}}).\end{align*}

Subsequently, we continue to use the features without undergoing the filtration, referred to as the original features. We perform recombination learning using the filtered features $\mathcal{D}_{{sc}^{\triangle }}$. We utilize unfiltered scRNA-seq features as query values. We reassemble the input scRNA-seq features using the filtered gene expression features, aiming to utilize the streamlined gene expression for better imputation of target ST data. The specific formula is expressed as follows: 


(10) \begin{align*}& \mathcal{A}_{r}^{\triangle} = \text{softmax} (Q_{\mathcal{S}} \otimes K_{\mathcal{S}^{\triangle}} / \sqrt{\Lambda}) \otimes V_{\mathcal{S}^{\triangle}} + \lambda \mathcal{D}_{{sc}^{\triangle}}.\end{align*}

By identifying and selecting marker genes or other informative genes, the module prioritizes genes that contribute positively to spatial reconstruction. This process excludes genes with negative correlations, which might otherwise introduce noise or irrelevant information. The adaptive filtration factor $\phi $ provides flexibility in gene selection, allowing the model to dynamically adjust the proportion of selected genes based on their contributions. This adaptability ensures that the model remains robust across different datasets and biological contexts. By recombining the filtered gene expression features with the original unfiltered features, the model leverages both the detailed information from selected genes and the broader context of the complete gene set. This balanced approach enhances the model’s overall performance in reconstructing spatial gene expression patterns. By recombining the filtered gene expression features with the original unfiltered features, the model leverages both the detailed information from selected genes and the broader context of the complete gene set. This balanced approach enhances the model’s overall performance in reconstructing spatial gene expression patterns.

### Validation benchmarks and strategy

We selected datasets from three different biological systems to validate the feasibility of the proposed method: drosophila embryo, mouse cortex tissue, human breast cancer tissue, and mouse brain anterior tissue:

(i) The single-cell resolution spatial dataset of drosophila embryos [20] encompasses spatial data marked on 3039 locations per cell with 84 genes, while the scRNA-seq data was detected in 1297 cells with 8924 genes.
(ii) For the mouse cortex tissue, the Smart-Seq dataset [21] comprises 15 413 cells and 45 768 genes from the Primary Visual Cortex (VISp) in a mouse brain slice. The corresponding single-cell resolution spatial atlas is obtained from various spatial transcriptomics technologies, including multiplexed error-robust fluorescence in situ hybridization (MERFISH) [22], and spatially resolved transcript amplicon readout mapping (STARmap) [23]. The scRNA-seq data in MERFISH contains 2399 cells and 254 genes, with 254 genes overlapping with the corresponding spatial data. The scRNA-seq data in STARmap contains 1549 cells and 1020 genes, of which 996 genes overlap with the corresponding spatial data.
(iii) The spatial human breast cancer tissue was obtained from the 10x Genomics data repository https://www.10xgenomics.com/resources/datasets/human-breast-cancer-block-asection-1-1-standard-1-1-0. The corresponding scRNA-seq data were downloaded from the GraphST repository [18]. The spatial data contains 3798 spots with 36 601 genes and the processed scRNA-seq data consists of 46 080 cells with 5000 genes, of which 921 genes overlap with the corresponding spatial data.
(iv) The mouse brain anterior (MBA) dataset was manually annotated with 52 regions using the Allen Brain Atlas reference [https://mouse.brain-map.org/static/atlas]. The corresponding scRNA-seq data were downloaded from the GraphST repository [18]. The spatial data in MBA contains 2695 spots with 32 285 genes, and the processed scRNA-seq data consists of 116 921 cells with 22 764 genes. By taking the union of the two datasets, we identified 1099 overlapping genes.

In terms of validation strategy, we referred to the approach used by Tangram [21], applying a K-fold cross-validation from the perspective of genes to quantitatively and visually validate the effectiveness of the methods. For each benchmark, we used a fixed parameter of K=10 for training and testing all methods.

### Comparisons methods

To ensure a fair comparison, we evaluated the state-of-the-art methods in the field, specifically STEM [24], GraphST [18], SpaOTsc [16], NovoSpaRc [25], and Tangram [19]. Additionally, we included the representative imputation methods gimVI [26] and stDiff [27] in our comparison.

In our experiments, to ensure a more comprehensive and fair comparison, we selected five metrics to quantitatively analyze the final results: Pearson Correlation Coefficient (PCC), Spearman Rank Correlation Coefficient (SPCC), Structural Similarity Index Measure (SSIM), Root Mean Square Error (RMSE), and Cosine Similarity (COSSIM).

## Results

### SELF-Former captures true cellular topological structures in reconstruction results

**Figure 2:**
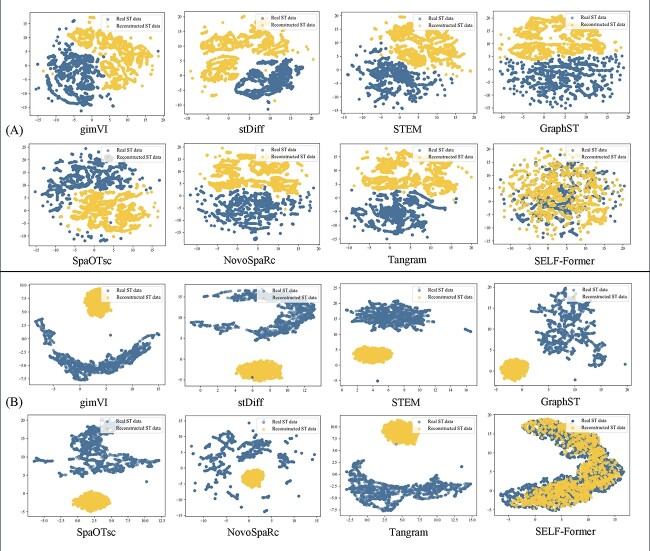
Comparison of various methods and SELF-Former on UMAP visualizations of real ST data and data after spatial reconstruction. (A)/(B) show the results on the Drosophila and STARmap. Yellow points denote spatial reconstruction data from scRNA-seq, while blue points represent the real ST data.

The objective of spatial reconstruction is to better match the distribution of ST data in the target domain. Specifically, we performed UMAP visualization of the ST data and corresponding scRNA-seq data to help readers understand the initial dataset distribution. We further visualized the results of seven comparison methods as well as SELF-Former, focusing on the reconstructed ST data using UMAP to determine whether they lie on the same distribution manifold. The specific visualizations are shown in Fig. 2.

Figure 2(A) presents the results on the Drosophila dataset. Since the Drosophila dataset was pre-processed beforehand, the original UMAP visualization shows a relatively coherent spatial state. However, it can be observed that the imputation method gimVI did not successfully predict the spatial locations of the ST data. Other methods also did not achieve the expected results in predicting the ST data. In contrast, the Tangram method partially aligned the recovered spatial positions, with some points distributed within the ST data. Our method effectively matched the real ST data distribution after reconstruction. In Fig. 2(B), the basic trend in STARmap dataset is more pronounced compared to the Drosophila dataset. Almost all comparison methods predicted the data into a subspace with tightly packed spatial distribution. SELF-Former accurately predicted the ST data and precisely recovered the spatial positions on the spatial manifold, aligning accurately with the ST data.

The excellent spatial recovery capability of SELF-Former is attributed to its network training mechanism and gene-wise constraints. SELF-Former learns the gene-wise distribution relationships of the target ST data, thereby constraining the predicted ST data distribution and ensuring that the reconstructed data aligns with the true ST data distribution. This strategy effectively reconstructs the scRNA-seq data into the ST data transformation, eliminating the misalignment between the two batches of data. Our model effectively removes batch effects between scRNA-seq and ST data, outperforming other spatial reconstruction and imputation methods. In summary, SELF-Former demonstrates the ability to correct batch effects under two different prior distributions, accurately reconstructing the predicted ST distribution with the true ST distribution.

### Accurate gene selection for training achieved by SELF-Former

**Figure 3:**
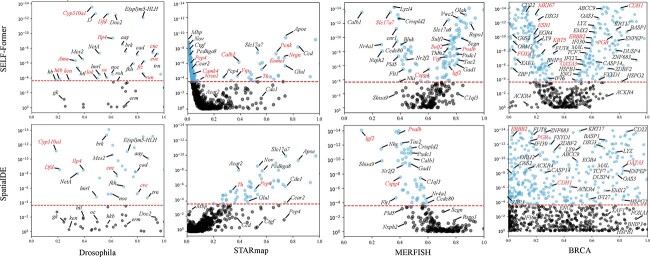
Scatter plots of gene selection on four benchmark datasets between SELF-Former and SpatialDE. The horizontal axis represents the fraction of variance of genes, while the vertical axis represents the Wilcoxon test $P$-values (log-transformed) calculated from predicted gene expression.

Gene selection is an essential module of our proposed SELF-Former. Existing research methods typically use the highly variable genes approach from the scanpy library as a preliminary selection method, selecting genes from thousands to tens of thousands of data points. This method introduces a certain degree of non-selectivity and lacks spatial characteristics. Some existing theoretical studies focus on spatial position-based selection methods, which are independent of reconstruction methods. To illustrate the significance of our proposed gene selection module, we provided comprehensive demonstrations of its diagnostic characteristics, as shown in Fig. 3.

For the significance of variant-selected genes, we plotted a scatter plot where the $x$-axis represents the Facction Variance (FV) score, and the $y$-axis represents the Wilcoxon test $P$-value (log-transformed) of the genes. The red horizontal dashed line indicates the significance threshold (FV = 0.001; -log p value). Genes above the red dashed line are considered significant for reconstruction results. Specifically, in the Drosophila dataset, genes marked in red are those with significant spatial positions after activation, such as Dfd, Mes2, and ftz, which exhibit distinct spatial patterns (striped or blocky). In the MERFISH and STARmap datasets, the red-marked genes are classic histological marker genes as indicated in [28]. In the BRCA dataset, the red-marked genes are those with clear spatial boundary positions[18, 29], eg. ERBB, CDH1, MKI67, KRT5, FOXA1, etc. It is evident that the number of genes surpassing Wilcoxon test $P$-values identified by SpatialDE is significantly fewer compared to those identified by SELF-Former. Moreover, SpatialDE shows a disadvantage in the selection of marker genes. For instance, notable genes such as ‘Camk4’ and ‘Vip’ in the STARmap are not identified by SpatialDE. This discrepancy arises because SpatialDE assumes that the spatial variation in gene expression is smooth and continuous. However, gene expression patterns often exhibit more complex nonlinear characteristics that Gaussian process models which SpatialDE relies on, may fail to capture effectively. Additionally, SpatialDE’s use of random effect terms to model non-spatial variability poses challenges in handling noisy data. High levels of noise can interfere with the identification of spatial variation components, thereby affecting the accuracy of the results. In contrast, SELF-Former employs a self-attention mechanism, which is more adept at capturing complex nonlinear spatial features. Compared to Gaussian process models, the self-attention mechanism can more flexibly adapt to various expression patterns, thereby enhancing the accuracy and comprehensiveness of gene selection. SELF-Former selects genes based on a reconstruction objective, which allows it to identify genes that are more beneficial for reconstructing spatial gene expression patterns. This objective-driven selection process effectively captures significant gene expressions that are crucial for accurate spatial reconstruction by ensuring a comprehensive and systematic approach to the analysis.

We also designed comparative experiments to demonstrate the efficiency and effectiveness of the gene filtering module, as shown in Table 1. We devised two metrics: Overlap Rate (OR) and validation time. The table presents a comparative analysis of gene filtration methods across four datasets: MERFISH, STARmap, Drosophila, and BRCA. The methods compared include SpatialDE, BayesSpace, and SELF-Former. The metrics used for comparison are the OR, which represents the percentage of overlap with highly variable genes, and the time taken for the gene filtration process in seconds. In MERFISH dataset, SELF-Former outperforms both with an OR of 25.23% and the fastest processing time of 0.19 seconds. Also, SELF-Former again demonstrates superior performance with an OR of 33.15% and a processing time of just 0.33 seconds in STARmap dataset. Additionally, on the BRCA dataset, we demonstrated performance improvements in both overlap rate and speed. SELF-Former showed a 26% (29.34%) improvement in the OR metric compared to SpatialDE (BayesSpace). In summary, SELF-Former consistently provides the highest overlap rates across all datasets while maintaining the fastest processing times, demonstrating its efficiency and effectiveness in gene filtration.

**Table 1:** Comparison of various gene filtration methods highlighting their overlapping rates and times across different datasets. The overlap rate is expressed as a percentage and indicates the effectiveness of each method in identifying common gene expressions. The processing time is measured in seconds, providing insights into the computational efficiency of each approach.

Methods	Drosophila		MERFISH		STARmap		BRCA	
	OR(%)$\uparrow $	Times(s)	OR(%)$\uparrow $	Times(s)	OR(%)$\uparrow $	Times(s)	OR(%)$\uparrow $	Times(s)
SpatialDE[30]	12.12	30.62	4.54	320.87	8.45	1025.23	9.45	1125.11
BayesSpace[31]	7.02	3.15	3.23	24.32	4.12	77.12	6.11	83.12
SELF-Former	**18.02**	**0.04**	**25.23**	**0.19**	**33.15**	**0.33**	**35.45**	**0.43**

### SELF-Former outperforms state-of-the-art models in spatial reconstruction task

**Figure 4:**
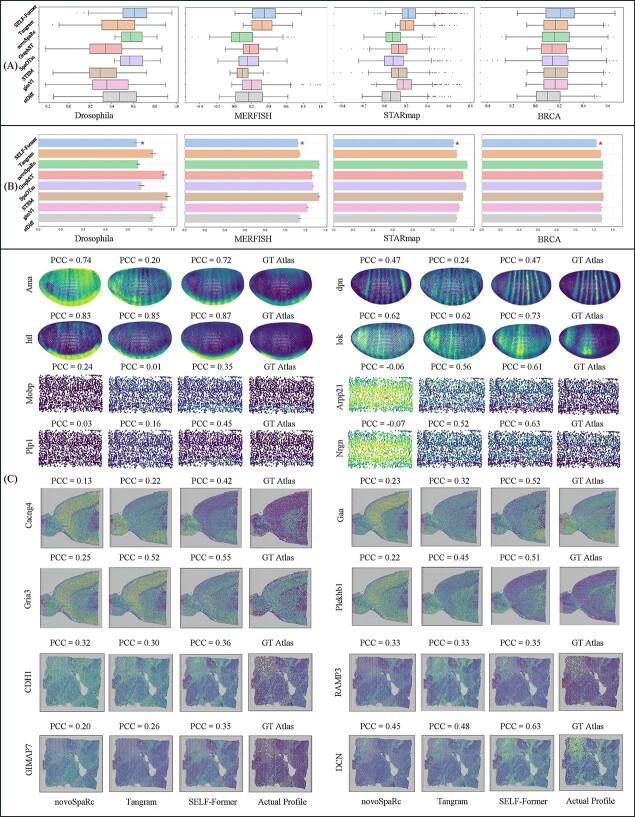
Quantitative and qualitative results of spatial reconstruction. (A) and (B) show the PCC box plots and RMSE bar charts on four datasets, respectively, for SELF-Former and seven comparison methods. (C) presents visualizations of genes with strong spatial localization in the Drosophila, STARmap, MBA, and BRCA datasets. We display images from SELF-Former, novoSpaRc, Tangram, and Actual Profile, with corresponding PCC values listed above each gene and gene names on the left side of each row.

Visualizations of the results and comparisons with other methods can be found in Fig. 4. Figure 4 (A) presents the box plots of PCC for various methods applied to the dataset. Each box plot illustrates the distribution of PCC values, with the box representing the interquartile range (IQR) and the whiskers extending to 1.5 times the IQR from the lower and upper quartiles. Outliers are shown as individual points outside the whiskers. SELF-Former exhibits the highest median PCC, indicating strong performance in correlating the predicted values with the true values. It also shows a relatively compact distribution of PCC values, suggesting consistent performance. Tangram and novoSpaRc follow with slightly lower median PCC values, while GraphST, SpaOTsc, and STEM show more variability in their PCC distributions. gimVI and stDiff display the lowest median PCC values, highlighting their relatively weaker correlation performance compared to other methods.

Figure 4(B) shows horizontal bar plots comparing the RMSE for the same set of methods. The mean RMSE values are represented by the length of the bars, with error bars indicating the standard error of the mean. The methods are ordered in the same sequence as in Fig. 4 (A) for consistency. SELF-Former demonstrates the lowest RMSE, depicted by the shortest bar, which signifies its superior performance in minimizing the prediction error. A red star marker highlights this method as the best performer in terms of RMSE.

In Fig. 4(C), we illustrate the spatial position reconstruction effects on Drosophila, STARmap, MBA, and BRCA datasets. The MBA and BRCA datasets were derived from 10x Genomics, with corresponding tissue images provided as references. SELF-Former effectively reconstructs the spatial expression of genes. For example, in the Drosophila dataset, the reconstruction of strip genes and prominently expressed bottom genes is particularly accurate. In the STARmap dataset, SELF-Former significantly outperforms Tangram and novoSpaRc in reconstructing genes such as Mobp and Plp1, which are prominently expressed at the bottom positions. In the MBA and BRCA datasets, SELF-Former demonstrates superior accuracy in the spatial positioning of genes. The reconstructed patterns align closely with the ground truth atlas, particularly in regions with significant expression. In contrast, Tangram and novoSpaRc lack spatial location priors for individual gene reconstructions.

Further, Table 2 reveals that SELF-Former consistently outperforms other methods across these metrics. For instance, in the MBA dataset, the average cosine similarity achieved by SELF-Former is 0.5531, significantly higher than the second-best method. This superior performance can be attributed to the effective gene selection and spatial position constraint losses we introduced. These enhancements enable SELF-Former to maintain excellent numerical performance while accurately reconstructing the spatial expression patterns of genes.

**Table 2:** Quantitative results between real ST data and reconstructed ST data by Tangram, novoSpaRc, GraphST, SpaOTsc, STEM, gimVI, and SELF-Former on Drosophila, MBA, and BRCA.

Datasets	Drosophila			MBA			BRCA		
	SPCC$\uparrow $	SSIM $\uparrow $	COSSIM$\uparrow $	SPCC$\uparrow $	SSIM $\uparrow $	COSSIM$\uparrow $	SPCC$\uparrow $	SSIM $\uparrow $	COSSIM$\uparrow $
Tangram	0.3641	0.3687	0.8231	0.1754	0.0998	0.4882	0.1679	0.0530	0.4144
novoSpaRc	0.2155	0.1884	0.0812	0.1544	0.0648	0.3884	0.1468	0.0015	0.4140
GraphST	0.2683	0.2483	0.8014	0.1598	0.0544	0.3845	0.1995	0.0045	0.3937
SpaOTsc	0.3936	0.4402	0.7819	0.1665	0.1002	0.4021	0.1469	0.0156	0.4119
STEM	0.2838	0.2483	0.6657	0.1845	0.1021	0.4687	0.1685	0.0531	0.4156
gimVI	0.3393	0.1058	0.8165	0.1645	0.1121	0.4517	0.1682	0.0532	0.4161
SELF-Former	**0.4687**	**0.4830**	**0.8328**	**0.2273**	**0.1581**	**0.5531**	**0.2048**	**0.1388**	**0.4877**

### Ablation study and implementation details

To validate the effectiveness of our proposed model architecture and regularization constraints, we introduced four corresponding variants and evaluated their performance on the STARmap and MERFISH datasets. The results are summarized in Table 3.

- Module-Variant-1: removal of the multi-scale module;
- Module-Variant-2: removal of the gene filtration learning;
- Loss-Variant-1: removal of $\mathcal{L}_{recon}$ and $\mathcal{L}_{mask}$;
- Loss-Variant-2: removal of $\mathcal{L}_{corr}$.

**Table 3:** Quantitative results of various model variants evaluated on STARmap and MERFISH datasets, focusing on performance metrics including PCC, SPCC, SSIM, RMSE, and COSSIM. These metrics provide a comprehensive assessment of the models’ ability to accurately reconstruct spatial gene expression patterns.

Variants	STARmap					MERFISH				
	PCC$\uparrow $	SPCC $\uparrow $	SSIM$\uparrow $	RMSE$\downarrow $	COSSIM$\uparrow $	PCC$\uparrow $	SPCC $\uparrow $	SSIM$\uparrow $	RMSE$\downarrow $	COSSIM$\uparrow $
Module-Variant-1	0.2099	0.1845	0.0842	1.2722	0.4015	0.3125	0.2514	0.2015	1.1615	0.4215
Module-Variant-2	0.2155	0.1884	0.0812	1.2451	0.4154	0.3348	0.2678	0.2248	1.1443	0.4412
Loss-Variant-1	0.1654	0.1654	0.0611	1.2978	0.3889	0.2484	0.1814	0.1872	1.2311	0.3548
Loss-Variant-2	0.1933	0.1789	0.0074	1.2845	0.3945	0.2778	0.2019	0.0187	1.2102	0.3784
SELF-Former	**0.2359**	**0.2330**	**0.1016**	**1.2327**	**0.4387**	**0.3566**	**0.2780**	**0.2493**	**1.1270**	**0.4692**

The performance metrics used for evaluation include PCC, SPCC, SSIM, RMSE, and COSSIM. For the STARmap dataset, SELF-Former achieves the highest PCC (0.2359), SPCC (0.2330), SSIM (0.1016), COSSIM (0.4387), and the lowest RMSE (1.2327), demonstrating superior performance compared to other variants. The other methods show varying levels of performance, with Module-Variant-2 performing better than Module-Variant-1 and Loss-Variant-2 performing better than Loss-Variant-1 across most metrics. Similarly, for the MERFISH dataset, our method also performs best, achieving the highest PCC (0.3566), SPCC (0.2780), SSIM (0.2493), COSSIM (0.4692), and the lowest RMSE (1.1270). Removing $\mathcal{L}_{recon}$ and $\mathcal{L}_{mask}$ directly leads to a decrease in performance (PCC values) by 29.9%/30.3% in STARmap/MERFISH datasets. Regarding the gene-wised spatial correlation loss $\mathcal{L}_{corr}$, the absence of $\mathcal{L}_{corr}$ significantly reduces the predicted SSIM scores, decreasing by 0.0942 (92.7%) and 0.2306 (92.5%) in STARmap and MERFISH datasets, respectively. In summary, the proposed method demonstrates superior performance across both datasets, confirming its robustness and accuracy compared to the evaluated variants.

We summarized the basic architecture of the network for the all datasets in Table 4. All components of the network were jointly trained from scratch. To optimize the SELF-Former, we employed the Adam optimizer, known for its efficiency in managing sparse gradients, which are prevalent in high-dimensional gene expression data. We used the default momentum parameters, $\beta _{1} = 0.5$ and $\beta _{2} = 0.999$, to stabilize the training process and accelerate convergence. The optimization strategy involves gradient descent updates for each loss parameter, ensuring that the model learns effectively across different datasets. Additionally, we implemented an Exponential Moving Average of the model parameters. This technique helps smooth the parameter updates and enhances the stability and performance of the model during training. By combining these strategies, we achieved a robust optimization process that contributed significantly to the model performance.

**Table 4:** Overview of hyper parameters utilized across different datasets, including learning rates, the number of epochs, and specific parameter values.

Hyper parameters	Datasets				
	Drosophila	MERFISH	STARmap	BRCA	MBA
Learning rate	1e-5	5e-5	5e-5	5e-5	5e-5
Epoch	180	400	800	400	1000
Parameters $\alpha _{1}$	0.1	0.1	0.2	0.2	0.2
Parameters $\alpha _{2}$	0.5	0.5	0.5	0.5	0.5

### The architecture contributes to improving the efficiency and performance of spatial reconstruction

**Figure 5:**
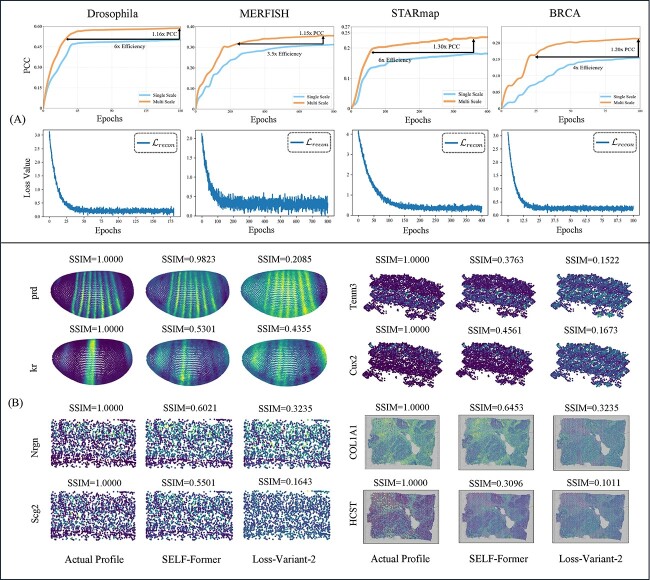
Network structure efficiency analysis curves and visualization of spatial position correlation constraints. (A) shows two sets of curves. The first set compares the model performance and efficiency improvements for multi-scale and single-scale structures, with the vertical axis representing the corresponding PCC values and the horizontal axis representing the number of training epochs. The second set displays the reconstruction loss $\mathcal{L}_{recon}$ curves, illustrating the decrease in loss values over training epochs. (B) presents visual comparison images with actual profile, SELF-Former and w/o loss $\mathcal{L}_{corr}$.

We conduct an evaluation to assess the impact of the multi-scale strategy in the spatial reconstruction task. As shown in Fig. 5(A) top, we compare the single-scale transformer and multi-scale transformer in terms of training efficiency and performance gain in four benchmarks. The results clearly demonstrate that the implementation of the multi-scale strategy significantly enhances the reconstruction process, both in terms of efficiency and performance. This highlights the effectiveness of incorporating multi-scale strategy within proposed model. Further, by minimizing the mean squared error loss $\mathcal{L}_{recon}$, SELF-Former achieves a global estimation of the predicted output, ensuring that the overall gene expression patterns are accurately reconstructed. Figure 5(A) shows a downward trend of $\mathcal{L}_{recon}$ converges and tends to $0$, proving that SELF-Former retains critical information well during training. Simultaneously, employing $\mathcal{L}_{mask}$ facilitates the estimation of non-zero elements, enabling SELF-Former to effectively reconstruct the spatial locations of scRNA-seq data. This enhancement boosts the model’s ability to encode scRNA-seq data, capturing both intrinsic and extrinsic gene expression patterns. Consequently, it improves the integration and prediction accuracy of spatially resolved reference data.

In Fig. 5(B), we focus on the genes prd, kr, Tncm3, Cux2, Nlgn, Sgc2, COL1A1, and HCST to analyze the significance of the correlation loss $\mathcal{L}_{corr}$ in maintaining spatial structure and improving model performance. Four benchmarks demonstrate that the SSIM performance of SELF-Former, which includes the correlation loss, retains a high degree of spatial structure. The SSIM values in the loss-variant model show a significant reduction, highlighting the importance of correlation loss in retaining spatial information. The inclusion of gene-wise correlation loss $\mathcal{L}_{corr}$ plays a crucial role in maintaining the spatial integrity of gene expression patterns across various datasets. The substantial differences in SSIM values between our model and the loss-variant model underscore the effectiveness of $\mathcal{L}_{corr}$ in enhancing spatial fidelity. This analysis demonstrates that correlation loss significantly contributes to preserving the intricate spatial structures of gene expression, which is vital for accurate ST data analysis.

### SELF-Former preserves the correlation of the reconstructed data both within and between datasets

**Figure 6:**
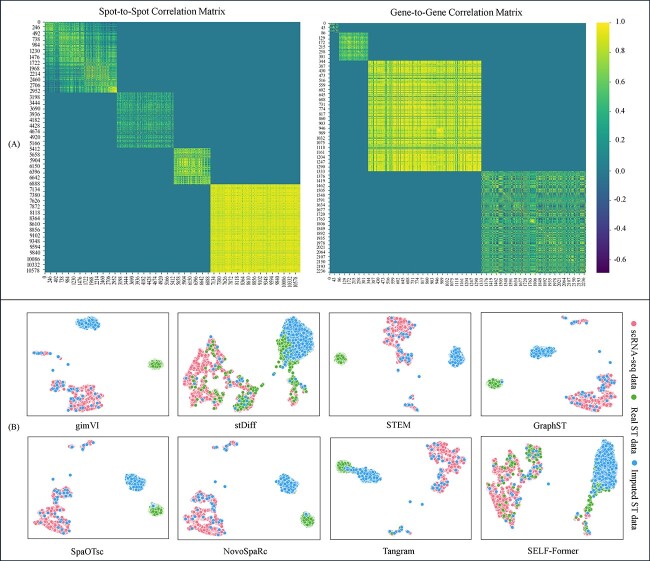
(A) Heatmap visualization of spot-spot and gene-gene correlations across different benchmarks. The axes represent spots/genes indices from the datasets: Drosophila, MERFISH, STARmap, and BRCA. (B) The clustering analysis of imputed data using different methods.

SELF-Former demonstrates outstanding modeling capabilities for spatial reconstruction tasks. It efficiently encodes and decodes scRNA-seq data through transformer blocks to achieve target ST data. Although we have previously validated that our method effectively corrects batch effects, possesses excellent gene selection capabilities, and achieves optimal performance, the fundamental significance of recovering ST data still requires discussion. For the spatial reconstruction results of each dataset by SELF-Former, we visualized the correlation matrices at both the spot and gene levels. Figure 6(A) visualizes the correlation matrices between the Reconstructed ST data and the Real ST data. The left panel presents the spot-to-spot correlation matrix, while the right panel shows the gene-to-gene correlation matrix. These matrices highlight high within-dataset correlations and lower between-dataset correlations, clearly specifying the relationships within and between the reconstructed and real data. Regarding the BRCA dataset, the observed high spot-to-spot correlation and relatively low gene-to-gene correlation are indeed due to its resolution, which is at 10x rather than single-cell resolution. The resolution impacts these correlations because at 10x resolution, each spot captures the averaged expression of multiple cells. This averaging process inherently increases the similarity between spots because variations at the single-cell level are smoothed out. Consequently, the spot-to-spot correlation appears higher because it reflects a more aggregated signal. Conversely, gene-to-gene correlations are lower because the detailed variations in gene expression within individual spot are lost in the averaging process, leading to a less precise capture of the gene expression patterns. This explains why the BRCA dataset at 10x resolution shows high spot-to-spot correlation but lower gene-to-gene correlation.

### SELF-Former performs well in ST imputation downstream task

We also investigated the application of reconstructed ST data for downstream tasks, particularly the imputation of ST data. We employed the reconstructed ST data in the recovery task, using stDiff as a benchmark framework. Specifically, we integrated the reconstructed ST data as conditional inputs into the stDiff model, sourced from various comparison methods, including SELF-Former, and utilized AdaLN to incorporate recovery information into the stDiff model. The performance of each model was assessed based on this integration. Figure 6(B) presents a UMAP visualization of scRNA-seq data, real ST data, and imputed ST data generated by stDiff, conditioned on Tangram, novoSpaRc, GraphST, SpaOTsc, STEM, gimVI, and the reconstructed ST data from SELF-Former based on STARmap. The results illustrate that stDiff’s predictions, conditioned on SELF-Former’s reconstructed ST data, closely align with the real ST data, while predictions based on other methods show significant deviations from the real ST data. Furthermore, using the reconstructed ST data from these methods as conditional inputs for downstream ST imputation tasks revealed a persistent batch gap, indicating discrepancies in spatial dimensions or shifts away from the actual ST data. In contrast, the reconstructed ST data from SELF-Former provided superior quality and more accurate spatial alignment, thereby enhancing the performance and interpretation of downstream tasks. This improvement highlights the superior quality of SELF-Former’s reconstructed ST data, which closely matches real data and mitigates batch effects, making it more suitable for various biological applications.

### SELF-Former can handle noise and variability in scRNA-seq data

SELF-Former incorporates several strategies to manage the inherent noise and variability in scRNA-seq data, ensuring robust and reliable performance. Normalization and Preprocessing: Prior to training, scRNA-seq data undergoes rigorous normalization and preprocessing to reduce technical variability and batch effects. This standardization minimizes the impact of technical noise, ensuring a more consistent input for the model. Self-Attention and Multi-Scale Mechanisms: SELF-Former leverages a self-attention mechanism to filter out noise by learning meaningful gene associations. This approach emphasizes significant gene relationships while suppressing spurious correlations. Additionally, multi-scale attention modules capture gene expression patterns across different scales, enhancing the model’s resilience to noise and variability. By combining these techniques, SELF-Former achieves a comprehensive and stable representation of gene expression data. Gene Filtration Module: A gene filtration module identifies and retains the most relevant genes for spatial reconstruction. Using an adaptive filtration factor, it selects genes with positive contributions, reducing the impact of noisy genes and improving the quality of reconstructed spatial data. Loss Functions: carefully designed loss functions play a crucial role in managing noise. The reconstruction loss ensures accurate capture of gene expression patterns, while mask loss focuses on estimating non-zero elements. The gene-wise correlation loss aligns predicted data with target spatial distributions, guiding the model to produce biologically meaningful and noise-resilient outputs. By integrating these strategies, SELF-Former effectively mitigates the effects of noise and variability in scRNA-seq data, producing robust and accurate spatial reconstructions.

### Ethical considerations and limitations of applying SELF-Former in clinical settings

The application of SELF-Former in clinical settings raises important ethical considerations and potential limitations that must be addressed to ensure responsible and effective use. Below, we outline several key points as follows:

1. Data Privacy and Confidentiality: clinical datasets often contain sensitive patient information. Ensuring the privacy and confidentiality of such data is paramount. This requires robust data anonymization and encryption methods to prevent unauthorized access and breaches.
2. Bias and Fairness: the quality and representations of the training data can significantly impact the performance of SELF-Former. If the training data is biased, the model may produce biased results, potentially leading to disparities in clinical outcomes across different patient groups.
3. Accountability and Transparency: in clinical settings, it is important for healthcare professionals to understand and trust the decisions made by AI models. SELF-Former should be designed to provide interpretable and transparent results, allowing clinicians to understand the basis of its predictions.

While SELF-Former holds significant potential for enhancing clinical decision-making through advanced spatial data reconstruction, it is crucial to address the ethical considerations and limitations associated with its application in clinical settings. Ensuring data privacy, mitigating biases, providing model transparency, and undergoing rigorous validation are essential steps to ensure that SELF-Former can be used responsibly and effectively in healthcare.

## Conclusions

In this work, we propose the SELF-Former framework for the task of spatial transcriptomics reconstruction from scRNA-seq data. SELF-Former is a transformer-based framework specifically designed for single-cell data spatial localization recovery tasks. It integrates gene expression feature learning with an attention mechanism to capture critical features of scRNA-seq data and corrects batch effects across different domains. Additionally, SELF-Former efficiently filters genes to select those that are meaningful for predicting ST data. The proposed network structure and spatial constraints have been thoroughly validated for their effectiveness and efficiency.

We evaluated the spatial localization recovery capabilities of SELF-Former across multiple datasets. SELF-Former outperformed existing spatial localization reconstruction and gene imputation methods both numerically and visually. Moreover, the correlation matrices between cells and genes further validated the reasonableness of the reconstructed data, suggesting that the biologically interpretable SELF-Former framework effectively models spatial reconstruction tasks and holds potential for downstream biological data analysis.

In the future, for the recovery of spatial positions using long gene sequences, we can enhance the model’s performance and interpretability by establishing gene-to-gene relationships and efficiently filtering out irrelevant genes. This approach holds potential value for the task of spatial localization recovery from scRNA-seq data and introduces a novel gene selection-based perspective to solve the spatial localization recovery task, offering significant room for expansion.

At the current stage, all models require training a new model for each different dataset to adapt to their respective spatial reconstruction tasks. It is not yet feasible to use a single model across all datasets. Future work could address this limitation by extending the application through transfer learning and domain adaptation, thus achieving more practical biological applications.

**Key Points**

- We propose a single-cell multi-scale gene filtration transformer structure for the spatially resolved reconstruction of scRNA-seq data. To the best of our knowledge, this is the first time a transformer-based framework has been utilized for modeling the intrinsic biological relationships between scRNA-seq data and ST data.
- Recovering the corresponding spatial positions of scRNA-seq data requires strong modeling capabilities. SELF-Former introduces attention and multi-scale modules to capture spatial features and mitigate batch effects.
- SELF-Former is the first to innovate by incorporating a gene filtration module in spatial recovery tasks, effectively filtering out less important genes in a task-oriented manner to enhance both model performance and efficiency.
- The performance of SELF-Former has been validated across multiple benchmark datasets, outperforming contemporary reconstruction, integration, and imputation methods in various metrics and visualization results. Our proposed method demonstrates significant potential value for downstream analysis.

## Data Availability

The implementation of SELF-Former is available at [https://github.com/bravotty/SELF-Former].
